# Multiple Kernel Attention Network for Dense and Tiny Wheat Pest Detection in the Field Under Complex Background

**DOI:** 10.3390/insects17070715

**Published:** 2026-07-10

**Authors:** Xiang Li, Mingqiang Chen, Lei Qian, Chenrui Kang, Kang Liu, Lin Jiao

**Affiliations:** 1 School of Computer Science and Artificial Intelligence, Chaohu University, Hefei 238000, China; 054087@chu.edu.cn; 2 School of Internet, Anhui University, Hefei 230601, China; mingqiangchen@stu.ahu.edu.cn (M.C.); lqian@stu.ahu.edu.cn (L.Q.); 3 School of Information Engineering, Southwest University of Science and Technology, Mianyang 621010, China; 4 Department of Aeronautical and Aviation Engineering, Hong Kong Polytechnic University, Hong Kong 999077, China; 5 Zhongke Hefei Institute of Technology Innovation Engineering, Hefei 230088, China

**Keywords:** wheat pest detection, grain aphids and mites, cut-up strategy, deep learning, MKA-Net, intelligent agriculture

## Abstract

The outbreak of pests seriously affects the yield and quality of wheat crops. The accurate recognition and detection play an essential role in the early warning of crop pests. While some limitations, like insufficient dataset, imbalanced samples of pests with dense distribution, and dense distribution and tinyof crop pests, pose significant challenges to the precise detection. Thus, in this work, we first spent two years collecting real-world wheat pest images with four types of pests, including three grain aphids, and one mite species, to obtain a high-quality crop pest dataset for network optimization. Secondly, to alleviate insufficiency of samples of pests with dense distribution, we have developed a cut-up data augmentation strategy that separates dense pest targets from complex backgrounds. Furthermore, to address the challenge of pest detection with tiny body size and dense distribution, we introduce the Multiple Kernel Attention Network (MKA-Net), which further integrates the multi-scale features of pests to improve detection accuracy. Our method achieved the best detection precision, with AP50 reaching its peak at 67.1%, which is a significant improvement of nearly 6.9 points in wheat pest detection compared with the baseline. In summary, our proposed method can assist in the prevention and control of wheat pests and promote the progress of intelligent agriculture.

## Simple Summary

Outbreaks of pest severely threatened wheat crop yields, yet accurate detection is often hindered by insufficient datasets and the tiny, dense nature of these pests. To address these challenges, this study establishes a high-quality, real-world dataset of wheat pests collected over two years. A cut-up data augmentation strategy was introduced to handle densely distributed targets, and the Multiple Kernel Attention Network was proposed to enhance the detection of small pests by integrating multi-scale features. The experimental results show that the proposed method achieves excellent performance for tiny and dense pest detection. Consequently, a valuable tool for early pest warning and improved crop protection is provided, which is expected to promote the advancement of intelligent agriculture.

## 1. Introduction

The occurrence of wheat pests can have severe negative impacts on agricultural production, the economy, and the ecological environment. For example, aphid, as a common agricultural pest, poses a serious threat to crop growth by feeding on plant sap, spreading viruses, and excreting honeydew, leading to colonization by pathogenic fungi and consequently to yellowing of leaves and even the death of the entire plant. Therefore, the precise detection of wheat pest is extremely important, which can help farmers quickly detect pest infestation and take targeted prevention and control measures, but also effectively reduce the excessive use of pesticides and reduce environmental pollution. In summary, early and timely wheat pest detection plays a crucial role in ensuring food security and promoting sustainable agricultural development, and is greatly significant for the management and development of intelligent agriculture.

With the emergence of Convolutional Neural Networks (CNNs) [1], the task of object detection achieved great success, and the field of object detection was pushed to a new level. Object detection modules based on CNNs are mainly categorized into two types: two-stage detection methods and one-stage detection methods. Two-stage object detection models are represented by the R-CNN series. Initially, Girshick et al. proposed the R-CNN, marking a new era for two-stage object detection models [2]. R-CNN employs a selective search algorithm to extract candidate regions, uses a convolutional neural network to extract features, and then applies a support vector machine for classification. Despite its effectiveness, R-CNN’s low speed and complex training procedure constrain its practical applications. Girshick et al. later refined the model and proposed the Fast Region-based Convolutional Network method (Fast R-CNN) for object detection, which incorporates Region of Interest, ROI pooling layers and a multi-task loss function, enhancing both efficiency and accuracy [3]. Subsequently, Girshick et al. further improved the model with the introduction of Faster Region-based Convolutional Network method (Faster R-CNN) for object detection, adding a region proposal network to enable end-to-end training of candidate regions, thus further boosting detection speed and target localization capabilities [4]. Following Faster R-CNN, He et al. extended the functionality of two-stage models with an advanced machine learning model for object detection and instance segmentation named Mask R-CNN [5]. Based on Faster R-CNN, Mask R-CNN adds a branch for generating instance segmentation masks, allowing the model not only to detect objects but also to precisely delineate their contours. The advent of Mask R-CNN has also led to the extensive application of two-stage object detection models in semantic segmentation tasks.

One-stage object detection models are exemplified by the you only look once (YOLO) series. Redmon et al. proposed YOLO, which transforms the object detection problem into a single regression problem, directly predicting bounding boxes and class probabilities on the image, thus achieving both localization and classification in a single forward pass [6]. Redmon et al. later introduced YOLOv2, which incorporates the concept of anchor boxes to improve the accuracy of bounding box predictions [7]. The module also adopts Batch Normalization and a new network architecture, further enhancing performance. In YOLOv3, Redmon et al. made structural improvements to the model, incorporating multi-scale predictions to enhance the detection of small objects, thus maintaining the speed advantage of YOLOv2 while improving accuracy [8]. Besides the YOLO family of one-stage detection models, other efficient one-stage models have emerged. For instance, Liu et al. proposed SSD, which performs detection on feature maps of various scales, improving detection accuracy and the ability to recognize small objects [9]. Tsung-Yi Lin et al. introduced RetinaNet, which addresses class imbalance with the Focal Loss, significantly enhancing detection performance [10].

Deep learning-based methods are widely applied for pest target detection. For example, Liu et al. proposed a region-based end-to-end method called PestNet, which integrates a new module channel space attention (CSA) into the backbone of a convolutional neural network (CNN) and replaces the fully connected (FC) layer with a position sensitive score map (PSSM) to reduce the number of parameters [11]. It performs well in multi-class pest detection. Jiao et al. combine the anchor-free region proposal network (AFRPN) and Fast R-CNN into one network to detect 24 types of pests, achieving excellent detection results [12]. Liu et al. used the YOLOv3 and adopted an image pyramid structure, fusing features from different levels to improve detection speed while ensuring detection accuracy [13]. Wang et al. proposed the Pest-D2DDet model, which has an efficient channel and spatial attention network (ECSA Net) architecture, and innovated an image preprocessing technique called Sparse Mask Super Resolution (SMSR) to form an automated pest recognition system that can identify 10 types of pests in natural environments [14]. To solve the problem of cotton pests and diseases detection, Zhang et al. improved YOLOX by introducing Efficient Channel Attention (ECA), hard-Swish activation function, and Focal Loss function, to enhance the ability of the model to extract image features, leading to the performance of detection speed and accuracy [15]. Dong et al. proposed a channel recalibration network for pest detection, including a channel recalibration feature pyramid network (CRFPN) and an adaptive anchor (AA) module. The CRFPN can effectively extract discriminative features to improve the accuracy of recognition and the ability to locate small pests; meanwhile, the AA module can correct the mismatch between anchor boxes and real bounding boxes, improving matching efficiency [16]. In order to better detect pests on citrus leaves, Qiang et al. used an improved Single Shot MultiBox Detector (SSD) model with a dual backbone network to detect citrus images. The experiment shows that the model exhibits high robustness on the citrus pest dataset [17]. Cheng et al. proposed a pest recognition method based on deep residual learning technology, and through optimization of deep residual learning, the recognition performance of this method has been significantly improved. When classifying crop pest images under 10 complex agricultural backgrounds, the accuracy rate reached as high as 98.67% [18]. Zhang et al. used a density map-based technique to identify and count multi-species pests to mitigate the negative impact of whiteflies and fruit flies on greenhouse crops. The experiment shows that their proposed method provides a new research path for counting and monitoring small target insect populations in dense images. Meanwhile, this also demonstrates the potential application of density map methods in various scenarios of small object detection [19]. Yang et al. proposed the multi-scale feature selection network (MFSPest), which designs a novel selective kernel spatial pyramid pooling structure (SKSPP) aimed at enhancing the network’s ability to extract features in key regions and reducing attention to irrelevant background information. MFSPest has reached the most advanced level in performance, with outstanding performance in accuracy, recall, and mean average precision (mAP) [20].

The limitations of current agricultural pest detection are mainly reflected in the following aspects:

(1) Insufficient dataset coverage and generalization: Many pest detection datasets have small sample sizes and poor generalization, making it difficult to accurately detect various types of crop pests. The proportion of pest objects in the dataset is too large in an image, which does not match the actual small size of pests. And the categories of pests in recent dataset are simple, making it difficult to cope with the diverse pest categories in practical scenarios.

(2) Sample imbalance and insufficient information: There exits an imbalance between foreground and background samples due to the small body size of pests, which will result in degradation of the detection module. At the same time, there is the serious imbalance between dense and sparse areas, which causes the poor performance of the pest detection network.

To address these problems, we first constructed a large-scale, small, and dense pest dataset, including 5685 images and 54,783 pest instances to address the problem of data deficiency. At the same time, we deeply explore the characteristics of this dataset, and propose a new cut-up strategy to increase the number of samples and alleviate the imbalance between dense and sparse areas. In addition, to address the challenge of detecting tiny and densely distributed pests, we introduce the Multiple Kernel Attention Network, which further integrates the multi-scale features of pests to improve detection accuracy. A large number of experiments show the effectiveness of the proposed method. The main contributions of this work are listed as follows:

(1) A high-quality wheat pest dataset has been constructed to address the issue of insufficient data. A total of 5685 images with four types (*Macrosiphum avenae*, *Sipha maydis*, *Penthaleus major*, and *Rhopalosiphum padi*) of pests have been collected and 54,783 pest instances are annotated.

(2) A cut-up data augmentation strategy to alleviate the challenges of insufficient samples and dense pest distribution. The proposed data augmentation strategy was developed to effectively separate dense pest targets from complex backgrounds.

(3) The Multiple Kernel Attention Network (MKA-Net) has been introduced to tackle the difficulty of detecting tiny and densely distributed pests (such as pests). It integrates multi-scale features to enhance detection accuracy, achieving a state-of-the-art AP50 (Average Precision at 0.50 Intersection over Union) of 67.1%, which represents a significant improvement of nearly 6.9 points over the baseline.

## 2. Related Works

### 2.1. Data Augmentation Strategy

To address the scarcity of small object samples, researchers have proposed various data augmentation strategies. Kisantal et al. introduced a copy–paste paradigm, where small objects are duplicated and randomly transformed within the same image to enrich training data [21]. Building on this, RRNet proposed AdaResampling, which leverages prior segmentation maps to guide valid position selection for pasting and incorporates scale transformations to mitigate size discrepancies [22]. Zhang et al. adopted divide-and-resize operations to amplify small object instances, enhancing sample diversity [23]. DS-GAN further advanced synthetic data generation by integrating object segmentation, inpainting, and blending techniques to produce high-fidelity small objects [24]. Beyond direct augmentation, optimized label assignment strategies (e.g., EMO [25]) have been explored to improve sample utilization by refining anchor matching or similarity metrics tailored for small objects. Despite progress, challenges remain in balancing augmentation effectiveness with computational overhead.

### 2.2. Small Object Detection

Recent advances in small object detection (SOD) focus on mitigating inherent challenges such as information loss, noisy features, and scale variation. Zhou et al. designed an efficient channel-spatial attention module and introduced a small object detection layer to improve the detection accuracy of small targets in the airport scene [26]. SCRDet devises an oriented object detector. In the detector, pixel attention and channel attention are trained under a supervised approach [27]. This training method serves to emphasize small object regions and simultaneously reduce the interference from noise. However, the computational cost of the attention module limits the real-time performance. Wu et al. introduced Self-Mimic Learning (SML) to align small object features with large ones, potentially suppressing unique characteristics of tiny targets [28]. Li et al. developed Perceptual GAN to generate high-resolution features via adversarial learning, but GAN instability may cause feature distortion [29]. Yuan et al. introduced Coarse-to-fine Region Proposal Network (CRPN), which ensures effective proposals for small objects through the dynamic anchor selection strategy and cascade regression [30]. Lin et al. proposed a Thick Film Ignitio module to build long-range dependencies between features of different scales through the self-attention mechanism, which fuses the multiscale feature representations from the encoder [31]. Wang et al. [32] designed a bidirectional feature pyramid network (BiFPN) to enhance the model’s ability to detect small objects. However, there is a lack of the ability to conduct subsequent feature processing. Therefore, for small object detection, it is still necessary to establish a connection between low-dimensional feature maps and high-dimensional feature maps.

## 3. Dataset

### 3.1. Data Acquisition and Annotation

**Figure 1:**
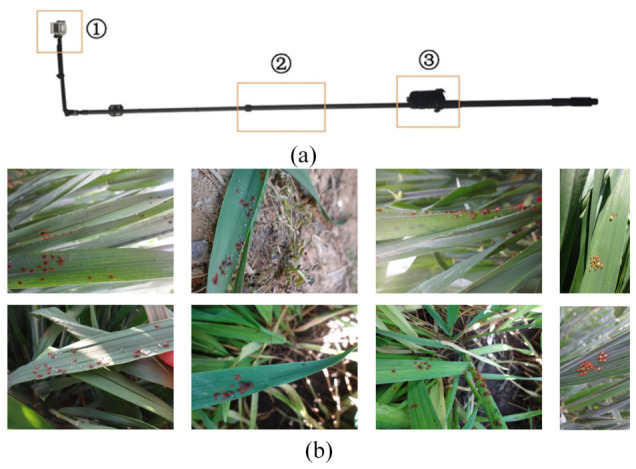
(**a**) is the collection device for capturing the pest iages; (**b**) shows some examples of pest images of wheat pest dataset.

Over the past two years, we have used a specially designed collection device to capture wheat pest images. Four different wheat pest species were registered: three grain aphids—Sitobion (Sitobion) avenae avenae (Fabricius, 1775) (*Macrosiphum avenae* (F.)) (MA), *Sipha maydis* Passerini, 1860 (SM), and *Rhopalosiphum padi* (Linnaeus, 1758) (RP), as well as blue oat mite *Penthaleus major* (Dugès, 1834) (PM). Some pest images from the collected dataset are shown in Figure 1. This device consists of a front-mounted macro lens camera, a mobile data transmission terminal, and a retractable carbon fiber support, as shown in Figure 1. The camera can easily penetrate deep into the wheat field for shooting and automatically upload the images to the server for storage and analysis. The resolution of images is about 1440 pixels × 1080 pixels. Images were captured under natural light conditions. To avoid harsh shadows and extreme lighting, data collection was conducted during specific time windows: from 8:00 a.m. to 11:00 a.m. and from 3:00 p.m. to 5:00 p.m. Given the small size of the wheat pests, the camera was positioned at a distance of approximately 10 cm from the target. Since the images were captured in a wild field environment, this distance was not strictly controlled; instead, the primary criterion was to ensure that the target pests were clearly visible and in focus. Four different types of wheat pest, namely *Macrosiphum avenae* (MA), *Sipha maydis* (SM), *Penthaleus major* (PM), and *Rhopalosiphum padi* (RP), have been collected. After obtaining the images of the wheat pests, the Labelme software V1.0 is adopted to annotate each pest in the images. To improve annotation consistency, a two-stage checking strategy was adopted. Each batch of images was first annotated by one annotator and then checked by another annotator. Inaccurate boxes and incorrect category labels were corrected after discussion. Samples with large annotation differences were annotated again. Finally, automatic scripts were used to check label files, including category indices, coordinate ranges, and invalid bounding boxes. The annotation information includes the center point coordinates (x,y) of the bounding box, the width *w* and height *h* of the bounding box, and the class name *l* of the pests. For pests that are severely occluded, if the visible part in the image is less than 20%, they are not annotated. The annotation files are saved in JavaScript Object Notation (JSON) format, which is similar to the format of the Common Objects in Context (COCO) dataset. Finally, the pests dataset contains 4291 images, with a total of 54,783 objects being wheat pests. To ensure a rigorous evaluation and prevent data leakage, the dataset partition was performed strictly at the level of original images prior to any preprocessing. The raw dataset was first divided into a training set of 3003 images and a validation set of 1288 images.

Table 1 demonstrates the number of pest images and pest instances. From Table 1 and Figure 1, we can conclude that the wheat pest dataset has the following characteristics: (1) various sizes of the pest images; (2) small size of the pest targets; (3) dense distribution of the pests; (4) long-tail distribution of the pest dataset. In the following section, we will give detailed analysis of these characteristics of the wheat pest dataset.

### 3.2. Data Analysis

**Figure 2:**
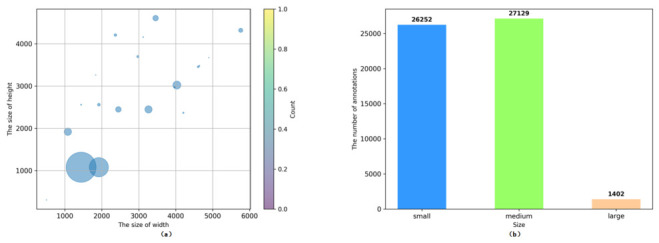
(**a**) is the size distribution of wheat pest in wheat pest dataset; (**b**) is the size distribution of pest instances in wheat pest dataset.

(1) Various sizes of the wheat pest image: We also counted the various image sizes in the wheat pest dataset, as shown in Figure 2a. The image sizes of this dataset are predominantly 1440 × 1080 and 1920 × 1080.

(2) Long-tail distribution of the wheat pest dataset: It is not difficult to see from Table 1 that the number of images for pest species ‘MA’ and ‘SM’ basically occupies the entire dataset, which is significantly larger than pest species ‘PM’ and ‘RP’. It can be seen that pest species ‘MA’ and ‘SM’ occupy the main distribution in the dataset. Table 1 also shows the number of instances of each category in the dataset. The number of instances for pest species ‘MA’ and ‘SM’ shows an overwhelming advantage, significantly greater than pest species ‘PM’ and ‘RP’. It can be seen that pest species ‘MA’ and ‘SM’ occupy the main distribution in the dataset. The deep learning-based modules underperform for the detection of wheat pest with few samples.

(3) Scale analysis of the wheat pest dataset: At the same time, according to the scale definition in MS COCO dataset, the bounding boxes of the wheat pest could be divided into three levels: small (area <32×32 pixels), medium (32×32 pixels < area < 96×96 pixels), and large (96×96 pixels < area). As shown in Figure 2b, the size distribution of each instance is concentrated in the small and medium categories. Furthermore, it can be seen that the scales of wheat pests are extremely small, resulting in poor performance for the pest detection module.

**Figure 3:**
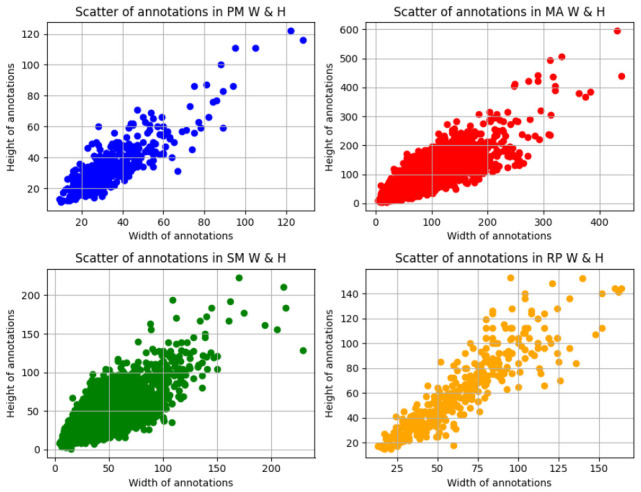
Distribution of width and height of bounding bounding boxes of four wheat pest species.

We have calculated the width (pixels) and height (pixels) of the bounding box of each pest species in this dataset. From Figure 3, it can be observed that the width and height of the bounding boxes for four wheat pest species ‘PM’, ‘MA’, and ‘SM’ show a clustering trend, whereas for the “RP” category they are evenly distributed. Additionally, we also note that the majority of the bounding boxes in pest species ‘PM’ have width and height smaller than 80 pixels. This demonstrates that the scales of the pest are various and tend towards tiny.

At the same time, we conducted a statistical analysis on the relative scale of all wheat pest targets in 4291 original images, and the relative scale of pests’ body size can be calculated as

(1) $$\mathrm{relative}\;\mathrm{scale}=\frac{h\times w}{H_{\mathrm{img}}\times W_{\mathrm{img}}}$$

where $H_{\mathrm{img}},W_{\mathrm{img}}$ and h,w are the height and width of the image and the bounding box, respectively.

**Figure 4:**
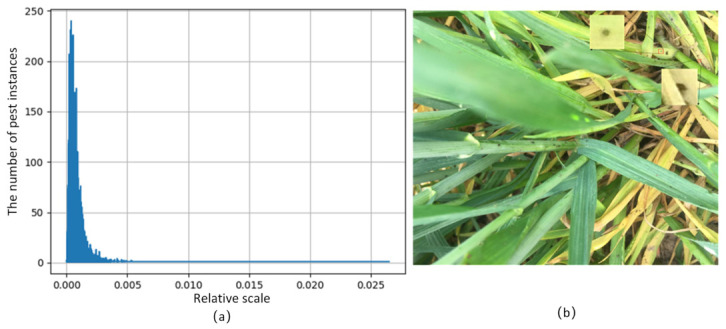
(**a**) is Relative scale distribution; (**b**) is a sample image from the dataset illustrating small-scale pest instances (Aphids).

Figure 4a plots the distribution of the relative scales of pest instances, showing that the relative scales of the pests are extremely small. For example, the relative scale only has 0.0018% of the most smallest wheat pest, as shown in Figure 4b, which brings considerable difficulty to the detection task.

**Figure 5:**
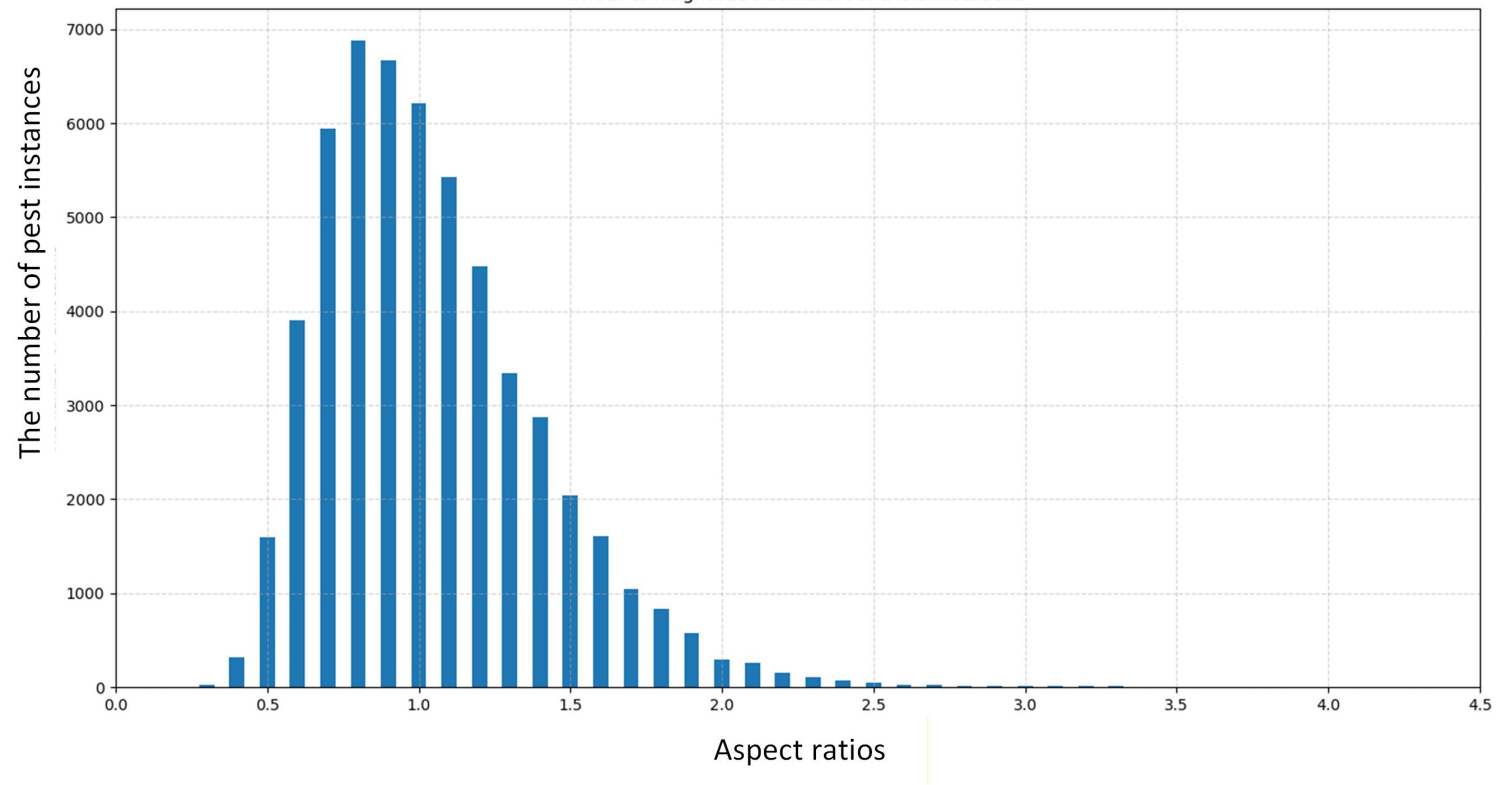
Distribution of aspect ratios of bounding boxes.

(4) Aspect ratio analysis of the wheat pests’ bounding boxes:

The aspect ratio affects the quality of bounding box regression. Further analysis of the aspect ratios of the wheat pest instances (Figure 5) demonstrates that the aspect ratios of the instances are various, which impedes the precise detection of the wheat pest. At the same time, for the anchor-based detectors, by combining the length and width sizes of the bounding boxes in Figure 4, we can provide help for designing anchor box sizes in the future.

**Figure 6:**
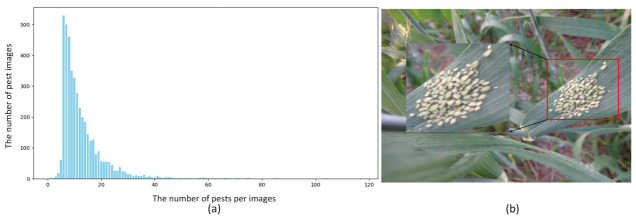
(**a**) is the distribution of the number of pest per image; (**b**) is a sample image from the dataset illustrating densely distributed pest instances (Aphids).

(5) Density analysis of the wheat pests dataset:

The denser the distribution of pests on each image, the greater the difficulty of detection. The number of pest instances in each image has been counted, as shown in Figure 6a. It clearly shows that the number of pest instances in each image is concentrated between 5 and 20. Figure 6b demonstrates the number of pest instances can up to be 86 in an image. It is not difficult to see from the figure that there is a serious occlusion and overlays, which increase the difficulty of detection.

## 4. Method

### 4.1. Cut-Up Strategy

**Figure 7:**
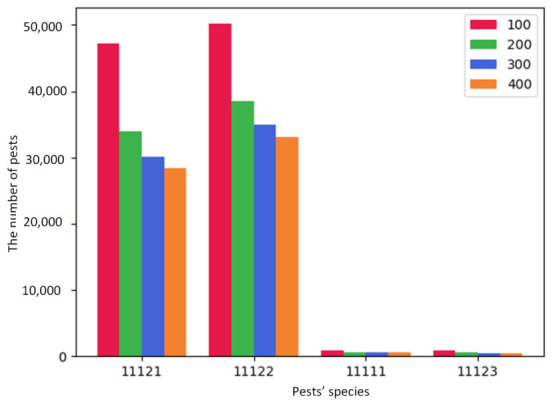
Category counts comparison.

By observing the wheat pest dataset, we can see that its distribution tends to be an aggregate distribution, possibly in clusters or bands, with scattered occurrences being less common. Furthermore, the backgrounds in our wheat pest dataset often appear complex, which poses numerous difficulties for the detection of wheat pests. This leads us to propose a hypothesis: could we cut-up the pest images into several parts to reduce the impact of cluttered backgrounds during detection? This would allow the model to focus more on the pests, thereby improving detection accuracy. Therefore, we proposed the cut-up strategy, and Algorithm 1 gives the pseudo-code. The cut-up strategy described above was applied exclusively to the samples in the training set. This protocol ensures that patches derived from the same original image do not appear in different sets, thereby eliminating the risk of the model memorizing background features across the training and validation phases. The cut-up operation involves cutting the wheat pest images into specified pixel sizes that are decided by the cut-up factor. Here, we set the cut-up factors to 100, 200, 300 and 400, respectively. Note that those generated samples without any pest instances will be discarded to reduce the impact of negative samples. Figure 7 visually presents the aggregation areas of the same number of instances under different pixel sizes. In the experiments section, we will discuss which pixel size is more suitable for the pest detection task.
**Algorithm 1:** Cut-up strategy
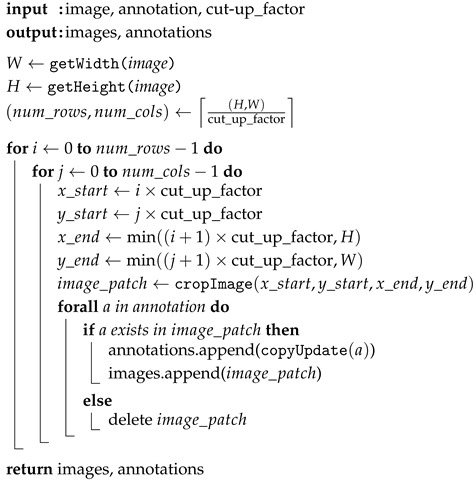


**Figure 8:**
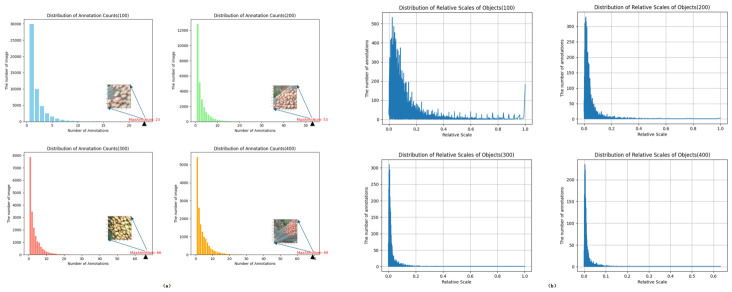
Statistical analysis of the number of instances and relative scales under different cut-up factors. (**a**) is a statistical comparison of the number of single instances under different cut-up factors. (**b**) is a comparison of relative scale distribution under different cut-up factors.

We have also conducted a comparison of the number of instances for each category after adopting the cut-up strategy. As shown in Figure 8a, when the cut-up factor is set to 100, the number of pest instances significantly increases compared to 200, 300, and 400. This is mainly due to the smaller pixel size for cut-up, which produces a larger number of pest images. However, a significant quantity of pest images contain repeated instances, which hinder the model training and bring about a negative influence on the prediction ability of the module. When the cut-up factors are set to 200, 300, and 400, the number of instances differs only slightly, indicating that 200, 300, and 400 may be more appropriate for image split.

**Figure 9:**
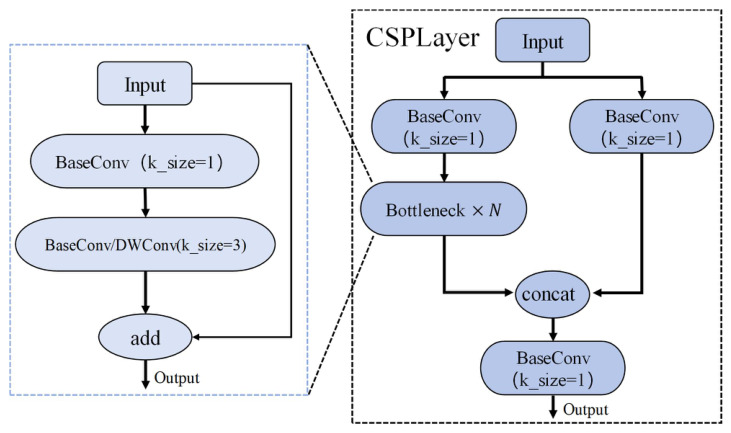
Structure of the CSPLayer.

In addition, we have counted the number of instances per pest image when using different cut-up factors, as shown in Figure 9. The number of instances per image increases with the cut-up factors, particularly during the transition from 100 to 200 and from 200 to 300 pixels. The change from 300 to 400, however, is not as pronounced. The reason for this phenomenon can be inferred from the characteristics of the wheat pest dataset, which include a greater consideration for dense distributions and regular patterns. Simultaneously, Table 2 shows the density distribution with different cut-up factors. It demonstrates that as cut-up factors increase, the number of instances per image also rises. This increase in the number of instances better reflects the true dense distribution of wheat pests, which is more in line with actual conditions. Consequently, this allows for a better understanding and learning of their distribution characteristics.

The relative scale distribution at different cut-up factors have been reported, as shown in Figure 8b. It shows that the relative scales of the pests tend to small no matter what the cut-up factor is. Furthermore, when the cut-up scale is 100, there are instances where the scale reaches 100%, indicating that some instances are overly large, occupying the entire image size. Additionally, it suggests that the pixel size might not have covered the entire instance, which is detrimental to learning the distribution of pests. As cut-up factor increases, the relative scale decreases more significantly.

### 4.2. Pest Detection Network

#### 4.2.1. Overall Architecture of the Pest Detection Module

**Figure 10:**
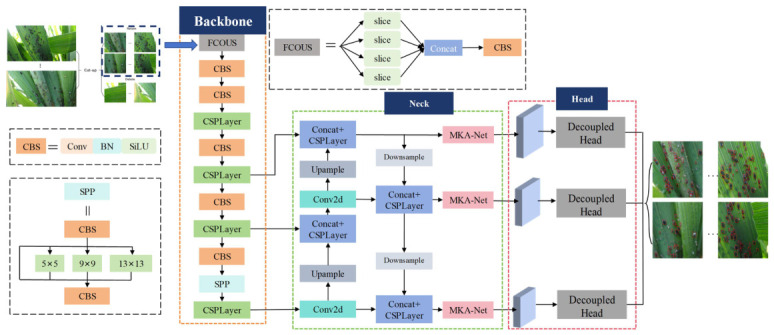
Overall structure of the pest detection module.

Figure 10 shows the overall architecture of the pest detection module. The input image undergoes cut-up data augmentation strategy to generate various samples. Then, the backbone network CSPDarknet is used to extract multi-scale features. Then, top-down and down-to feature pyramid networks are adopted, respectively, for obtaining multi-scale features. A lightweight attention module, Multiple Kernel Attention Network (MKA-Net), is designed for obtaining the feature maps of different scales, which aids in better extracting features of small targets under complex backgrounds and thus improves detection accuracy. Finally, the processed feature maps are fed into the detection head for regression bounding boxes and classification, yielding the final detection results.

#### 4.2.2. CSPDarknet Backbone

We utilized CSPDarknet as a backbone network, including Focus, CBS and CSPLayer. The input image is first fed into the Focus structure, which uses slicing operations to split high-resolution features into multiple low resolution feature maps. It generates four separate feature layers by extracting values from the input image at every other pixel. These four feature layers are then stacked together, integrating the width and height information of the image into the channel dimension, thus quadrupling the number of input channels. Consequently, the feature layers processed by the Focus structure change from the original three channels to twelve channels.

In the structure of CBS, we added a Batch Normalization (BN) layer after convolution. This ensures that the training speed is maintained as the network depth increases, allowing faster convergence. In addition, we employed the SiLU activation function, which is an improved version of both Sigmoid and ReLU. SiLU has the characteristics of being unbounded above and bounded below, smooth, and nonmonotonic. SiLU outperforms ReLU in deep models and can be considered a smooth version of the ReLU activation function.

The CSPLayer is a key feature extraction structure with a straightforward design, as shown in Figure 9. Essentially, it is a decomposition of traditional residual block stacking, divided into two parts: the main part still performs the original residual block stacking operations; the other part resembles a residual path, undergoing minimal processing before connecting directly to the end of the network. Thus, it can be said that a larger residual path is integrated into the CSP structure. Moreover, we introduced the Spatial Pyramid Pooling (SPP) structure before the last CSPLayer, utilizing pooling kernels of different sizes for maximum pooling operations to extract features, which effectively enhances the network’s receptive field.

#### 4.2.3. Dual Feature Pyramid Networks

The traditional feature pyramid network (FPN) enhances the model’s ability to detect multi-scale objects by upsampling and fusing multi-layer feature maps. In contrast, we adopt a Dual-FPN structure, which introduces two information flows (top-down and bottom-up) to further strengthen the multi-scale fusion of feature maps. This improves the network’s detection capability for objects of various sizes. Small objects often lose detailed information in low-resolution feature maps, and traditional FPN may not effectively recover this information. The Dual-FPN performs exceptionally well in small pest object detection, because the bottom-up information flow enhances lower-layer features, helping to capture fine-grained details of tiny pests. By enabling cross-scale information transmission, Dual-FPN facilitates effective fusion across multiple scales, significantly improving small object detection accuracy.

The specific implementation of Dual-FPN involves two information flows-top-down and bottom-up—that enhance the multi-scale fusion of feature maps. These two flows serve different purposes and interact at each layer of the network, improving the network’s adaptability to pests with various sizes.

The top-down information flow primarily starts from the higher layers of the network and progressively transmits information to the lower layers. The higher-level feature maps contain rich semantic information but have lower resolution. Through upsampling operations, these high-level semantic features are passed down to the lower-level feature maps. The feature maps obtained from the backbone network, starting from the lower layers and moving toward the higher layers, are denoted as $f_{i},\left(i=1,2,3\right)$. The mathematical expression for this operation can be written as:

(2) $$\begin{array}{cc} P_{5} & =\mathrm{Conv}(f_{3}) \end{array}$$

(3) $$\begin{array}{cc} P_{4} & =\mathrm{Conv}(\mathrm{CSPLayer}\left(\mathrm{UpSample}\left(P_{5}\right)⊕f_{2}\right)) \end{array}$$

(4) $$\begin{array}{cc} P_{3} & =\mathrm{CSPLayer}(\mathrm{UpSample}\left(P_{4}\right)⊕f_{1}) \end{array}$$

where $P_{i}$ (*i* = 5, 4, 3) respectively represent the three feature maps output from the top-down information flow, and ⊕ represents the fusion operation.

In the bottom-up information flow, the process starts from the lower-level feature maps and progressively transmits information to the higher-level feature maps. The lower-level feature maps have higher spatial resolution and can preserve fine-grained details. Passing information from bottom to top helps the higher-level feature maps capture more spatial information, thereby enhancing the detection ability for small objects.

In the bottom-up information flow, the lower-level feature maps undergo a downsampling operation and are fused with higher-level feature maps. This fusion helps supplement the fine details and improves the ability to capture small objects and fine-grained features. The mathematical expression for this operation can be written as:

(5) $$\begin{array}{cc} P_{3}^{\mathrm{out}} & =P_{3} \end{array}$$

(6) $$\begin{array}{cc} P_{i}^{\mathrm{out}} & =\mathrm{CSPLayer}(\mathrm{Downsample}\left(P_{i-1}-\mathrm{out}\right)⊕P_{i})\;i=4,5 \end{array}$$

where $P_{i}^{\mathrm{out}}$ respectively represent the final output feature maps obtained from the bottom-up information flow.

By employing a dual-channel feature pyramid network, we effectively reduce information loss and feature degradation. Within the dual-channel FPN, a comprehensive and accurate feature fusion is achieved through complementary information flows, both top-down and bottom-up, minimizing information loss and enhancing the model’s stability and reliability. This allows for more robust interaction between different scales, ensuring the integrity of features and the preservation of details.

#### 4.2.4. Multiple Kernel Attention Network (MKA-Net)

**Figure 11:**
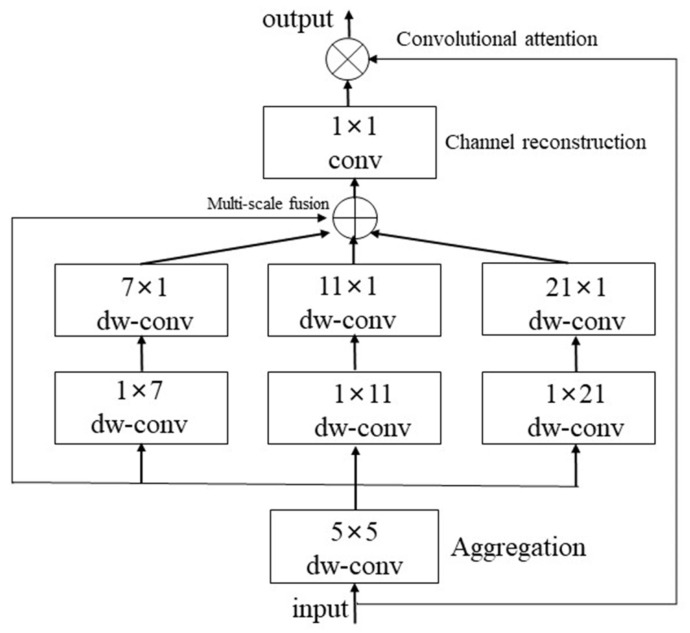
Illustration of the proposed MKA-Net.

Considering the effectiveness and efficiency of the pest detection network, we introduce an effective and simple Multiple Kernel Attention Network to further extract multi-scale identifiable features of pests. As described in Figure 11, MKA-Net consists of three parts: aggregation, extraction, and reconstruction. Firstly, a depth-wise convolution with kernel size of 5×5 has been used for aggregating the local information from the dual feature pyramid network. Secondly, multi-branch depth-wise strip convolutional layers (the kernel size of 7, 11, and 21 are used for large receptive field) are adopted for extracting multi-scale features. Finally, the relationships of different channels from multi-scale feature maps have been reconstructed by using the convolutional operation of 1×1. In our proposed pest detection module, three feature maps, $P_{3}^{\mathrm{out}}$, $P_{4}^{\mathrm{out}}$, and $P_{5}^{\mathrm{out}}$, generated from dual feature pyramid network are input to the MKA-Net for obtaining the classification and regression feature information. Mathematically, MKA-Net can be expressed as:

(7) $$\begin{array}{cc} \mathrm{Att} & ={\mathrm{Conv}}_{1\times 1}\left(\sum_{i=0}^{3}{\mathrm{Scale}}_{i}\left(\mathrm{DW}-\mathrm{Conv}\left(P\right)\right)\right),\\ \mathrm{Out} & =\mathrm{Att}⊗P \end{array}$$

Here, *P* refers to the input feature. Att and Out denote the attention map and the output result, respectively. The symbol ⊗ represents the element-wise matrix multiplication operation. DW-Conv is an abbreviation for depth-wise separable convolution. ${\mathrm{Scale}}_{i}$, where *i* takes values from {0, 1, 2, 3}, refers to the *i*-th branch in Figure 11. ${\mathrm{Scale}}_{0}$ represents the identity mapping. In each branch, we simulate a standard depth-wise separable convolution with a larger kernel size using two depth-wise separable strip convolutions. Specifically, the convolution kernel sizes for each branch are set to 7, 11, and 21. We choose depth-wise separable strip convolutions because strip convolutions have a lighter computational load. To mimic a standard 2D convolution of size 7×7, we only need to perform a combination of a 7×1 and a 1×7 convolution.

#### 4.2.5. Decoupled Pest Detection Head

**Figure 12:**
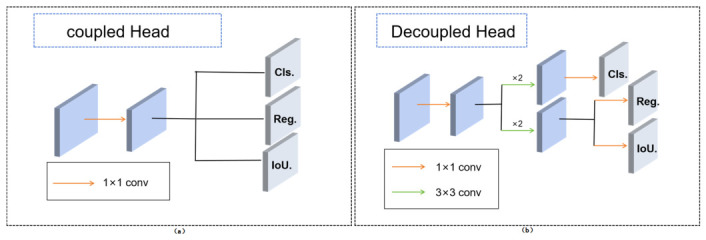
Description of difference between the coupled and decoupled detection heads. (**a**) is the coupled detection head and (**b**) is the decoupled detection head.

In object detection, the conflict between classification and regression tasks is a well-known problem, that is, classification and localization task are sensitive to different features. Thus, the decoupled head for classification and localization is widely used in most of the one-stage and two-stage detectors. Figure 12 depicts the difference between the coupled and decoupled detection heads. Similar to these detectors, we adopted the decoupled pest detection head, as shown in Figure 12b. The standard YOLO detection head has been replaced with a lite decoupled detection head. To be specific, it contains a 1×1 conv layer to reduce the channel dimension, followed by two parallel branches with two 3×3 conv layers, respectively, for classification and regression. Here, the classification branch outputs the classification score maps H×W×C and the localization branch outputs two prediction results. One is regression results of the bounding boxes, which determines the regression parameters at each point in the feature map, allowing for the adjustment of these parameters to obtain the predicted bounding boxes; the IoU branch is added on the regression branch to verify the precision of localization between the predicted bounding boxes and ground-truth bounding boxes.

### 4.3. Loss Function of Tiny Pest Detector

We employ CIoU [33] (Complete Intersection over Union) as our bounding box regression loss function. CIoU is an enhanced regression loss function for object detection bounding boxes, designed to refine the prediction of target bounding boxes with greater precision. In contrast to the conventional IoU loss, CIoU considers not only the overlap of bounding boxes but also incorporates factors such as the distance between boxes, their aspect ratios, and shapes, thereby enhancing the positioning accuracy of bounding boxes. The CIoU loss function can be formulated as follows:

(8) $$L_{CIoU}=1-IoU+\frac{\rho^{2}\left(b,b^{gt}\right)}{c^{2}}+\alpha v$$

where *b* and $b^{gt}$ represent the center points of the predicted and ground truth bounding boxes, respectively. $\rho^{2}\left(b,b^{gt}\right)$ is the squared Euclidean distance between the center points of the predicted and ground truth bounding boxes. *c* is the length of the diagonal of the smallest enclosing rectangle of the predicted and ground truth bounding boxes. α is a weight coefficient used to balance the influence of the aspect ratio.

*v* is a measure of the consistency of the aspect ratio between the predicted and ground truth bounding boxes, calculated as:

(9) $$v=\frac{4}{\pi^{2}}{\left(\arctan\frac{w^{gt}}{h^{gt}}-\arctan\frac{w}{h}\right)}^{2}$$

where *w* and *h* are the width and height of the predicted bounding box, $w^{gt}$ and $h^{gt}$ are the width and height of the ground truth bounding box.

The formula for calculating α is:

(10) $$\alpha =\frac{v}{\left(1-IoU\right)+v}$$

### 4.4. Evaluation Metrics

In the field of object detection, the PR (Precision–Recall) curve is a crucial evaluation tool. It measures a model’s performance by illustrating the relationship between precision and recall. During detection, the object detection model generates a bounding box for each potential target in an image. It also assigns a confidence score to each box, which indicates the probability that the box contains a target. To determine if a bounding box is correct, we commonly use IoU (Intersection over Union) as a metric. IoU is the ratio of the area of intersection to the area of union between the detection box and the ground truth box.

Precision is the proportion of detected boxes that actually contain a target among all boxes predicted as targets by the model. Its calculation formula is:

(11) $$\mathrm{precision}=\frac{\mathrm{TP}}{\mathrm{TP}+\mathrm{FP}}$$

Among these, TP (True Positive) represents the number of targets correctly detected, while FP (False Positive) represents the number of non-targets incorrectly detected as targets.

Recall is the proportion of all actual targets that are correctly detected by the model. Its calculation formula is:

(12) $$\mathrm{Recall}=\frac{\mathrm{TP}}{\mathrm{TP}+\mathrm{FN}}$$

FN (False Negative) represents the number of actual targets that the model failed to detect.

To plot the Precision–Recall (PR) curve, different confidence thresholds must be set, and the model’s outputs must be divided into targets and non-targets. For each threshold, calculate TP, FP, and FN to obtain precision and recall. Then, plot points on the PR graph with recall as the horizontal axis and precision as the vertical axis. Connect these points in order of recall to form the complete PR curve.

In object detection, mAP (mean Average Precision) is a widely used evaluation metric. It measures the model’s average performance across different target categories. The principle of mAP is based on calculating AP (Average Precision) for each category individually, and then averaging the APs across all categories. AP is the area under the Precision–Recall curve, which measures the model’s average precision across a series of recall rates.

The calculation of AP involves integrating the Precision–Recall curve. Typically, the following method is used to approximate AP: Divide the recall rates into several intervals (e.g., 0, 0.1, 0.2, …, 1.0), and within each interval, take the maximum precision. Calculate the average precision for each interval, which gives the AP. The formula can be expressed as:

(13) $$\mathrm{AP}=\sum_{r\in \{0,0.1,...,1.0\}}\left(\max_{\mathrm{recall}\geq r}\mathrm{precision}\right)\times \Delta r$$

where Δr is the width of the recall rate interval.

Next, we calculate mAP, which is the average of AP across all categories. First, calculate AP for each category individually, that is, AP is computed separately for each category in the dataset. Then, calculate mAP: average the APs of all categories to obtain mAP. The formula is:

(14) $$\mathrm{mAP}=\frac{1}{N}\sum_{i=1}^{N}AP_{i}$$

where *N* is the total number of categories, and $AP_{i}$ is the Average Precision for the *i*-th category.

We use the metrics mAP50 and mAP50:95 to measure the performance levels of different detection algorithms. mAP50 refers to the evaluation of the average precision across all categories when the Intersection over Union (IoU) reaches 0.5; mAP50:95, on the other hand, comprehensively evaluates the average precision for each category with IoU thresholds ranging from 0.5 to 0.95, in steps of 0.05. The higher the mAP value, the better the model’s average performance in detecting different categories of targets. mAP is particularly suitable for multi-category object detection tasks because it reflects the model’s overall performance across all categories, not just a single category. Additionally, mAP is an important evaluation metric for datasets with class imbalance, as it treats each category equally without giving higher weight to categories with more samples.

## 5. Experimental Results and Analysis

### 5.1. Comparisons of Different Object Detection Methods

We selected numerous classic target detection models, like Cascade-RCNN [34], Dynamic-Rcnn [35], Cascade-RPN, FCOS [36], TOOD [37], YOLOX [38], YOLOV5, YOLOV6 [39], YOLOV7 [40], YOLOV8 [41], and YOLOV10 [42], as comparison methods to verify the performance of the proposed tiny and dense pest detection module. Table 3 demonstrates the experimental results. It shows that our proposed module achieves the best results. From the perspective of detection accuracy, the proposed methods achieves 30.8% mAP, which obtains an improvement of 6 points compared to the suboptimal detector. In addition, when the IoU is set to 0.5 and 0.75, the mAPs are 67.1% and 22.0%, which are 5.1% and 8.9% higher compared to the suboptimal detection moduleYOLOV8. Furthermore, the mAPs reach 20.8%, 37.4%, and 39.8% for small, medium and large pest targets, respectively, obtaining improvements of 2.3, 8.5, and 1.1 points compared to the second best results. From the detection efficiency, Table 3 shows that the number of parameters and Giga Floating-Point Operations Per Second (GFLOPs) of our proposed module were 13.553 and 9.024M, which is slightly higher than the most efficient methods. Thus, the above experiments demonstrate that the proposed cut-up strategy and MKA-Net have the benefit of solving the problems of clustered and extremely small pest detection.

**Figure 13:**
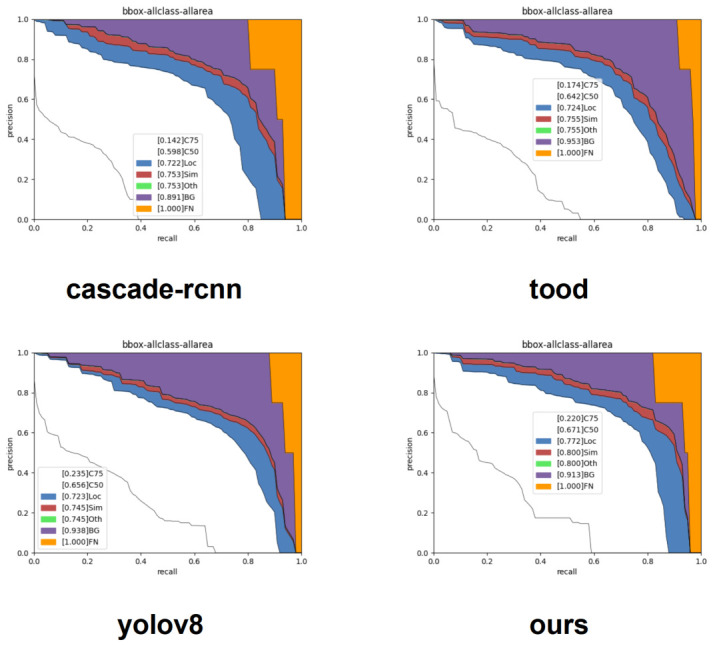
P-R curves of our method and other detection modules.

To comprehensively evaluate the performance of various models under different threshold settings, we plotted PR curves for Cascade-RCNN, TOOD, YOLOV8, and the proposed method, and compared them to gain a deeper understanding and comparison of their detection capabilities. Figure 13 reveal the balance between precision and recall for the models. It can be observed from the curves that our model achieved the highest accuracy of 0.671 at an IoU threshold of 0.5, and also demonstrated good performance at an IoU threshold of 0.75.

### 5.2. Visualization of Detection Results Using Different Methods

**Figure 14:**
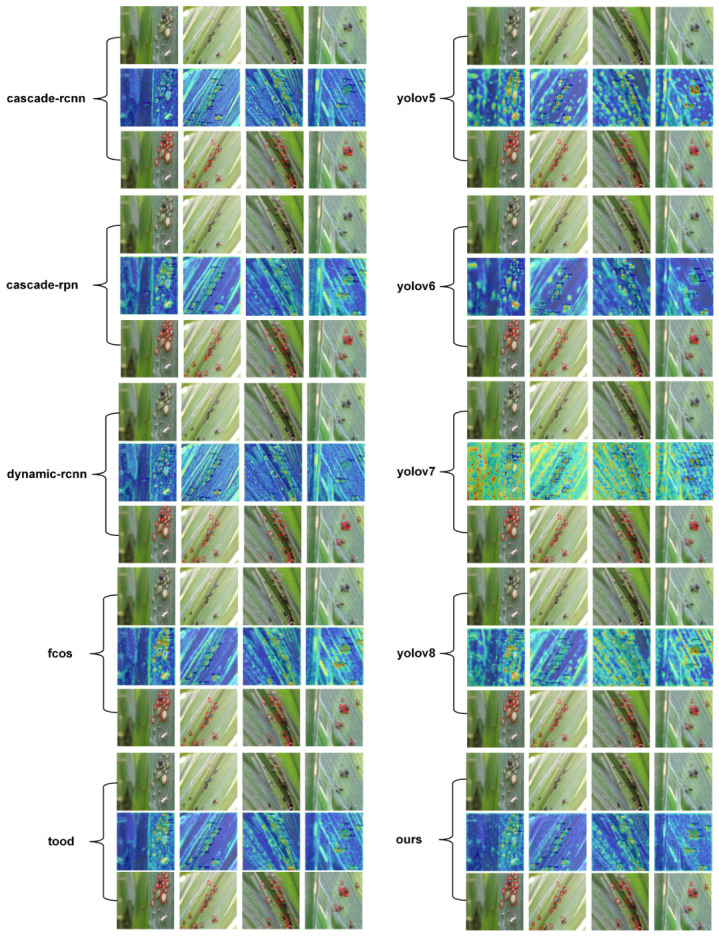
Visualization of heat maps for different detection models.

Figure 14 shows a large number of pest detection results among our methods and Cascade-RCNN, Dynamic-RCNN, Cascade-RPN, FCOS, TOOD, YOLOX, YOLOV5, YOLOV6, YOLOV7, and YOLOV8, and YOLOV10. The visualization of heatmaps offers an intuitive method, allowing us to clearly observe the areas of focus for different models when processing input images. This process enables us to deeply analyze whether the model’s attention is indeed concentrated on the target objects, without being distracted by the background or other irrelevant regions. As clearly demonstrated in Figure 14, our model effectively focuses on the target areas and successfully extracts key features, which is crucial for ensuring detection accuracy.

### 5.3. Performance of Cut-Up Strategy

To explore the role of the cut-up strategy in tiny and clustered pest detection task, we have conducted experiments without the cut-up strategy (only use the original pest datasets) and with the cut-up strategy. We choose Cascade-RCNN, Dynamic-RCNN, Cascade-RPN, FCOS, TOOD, YOLOX, YOLOV5, YOLOV6, YOLOV7, YOLOV8, and YOLOV10 as a baseline to fully demonstrate the effectiveness of the cut-up strategy. The results are shown in Table 4. The proposed cut-up strategy combined with Cascade-RCNN, Dynamic-RCNN, FCOS, TOOD, YOLOV5, YOLOV6, YOLOV7, YOLOV8, YOLOV10, and YOLOX surpass the original modules by 4.5 points (25.7% vs. 21.2%), 4.3 points (23.7% vs. 19.4%), 4.2 points (26.0% vs. 15.3%), 8.2 points (23.5% vs. 15.3%), 4.4 points (26.2% vs. 21.8%), 0.8 points (21.9% vs. 21.1%), 6.0 points (28.7% vs. 22.7%), 2.5 points (20.7% vs. 18.2%), 4.6 points (29.4% vs. 24.8%), 7.5 points (25.5% vs. 18.0%), and 6.7 points (30.2% vs. 23.5%), respectively. More importantly, the proposed cut-up strategy consistently outperforms all baseline methods with nearly 5 points improvement, demonstrating a significant enhancement during network training. Additionally, the detection performance for small and medium pest targets also significantly improves when using our proposed cut-up strategy. However, for large pest detection, the introduction of the cut-up strategy will hinder the detection of large pests. This may be because the cut-up strategy will split large pests into several patches, disrupting the integrity of the features of large pests. Furthermore, the results in Table 4 also indicate that the YoloX model performed the best. The YOLOX model achieved an mAP of 67.7% at 0.5 IoU, significantly outperforming other methods. When using stricter criteria, with IoU ranging from [0.5:0.95], it reached an mAP of 30.2%, which is a notable improvement over the results of the YOLOX model without the cut-up strategy.

### 5.4. Performance of MKA-Net

Considering the excellent performance of YOLOX, we select YOLOX model as a baseline to further verify the performance of the MKA-Net on clustered and tiny pest datasets, as shown in Table 5. It demonstrates that the cut-up strategy will significantly improve the performance of clustered and tiny pest detection. At the same time, we also are glad to find that the YOLOX combined with MKA-Net outperforms the original YOLOX by 6.7 points. Moreover, the introduction of MAK-Net will result in a performance gain of 7.3%. We also note that the introduction of the proposed MKA-Net only requires a slightly higher computation cost. For example, it only adds 0.231G Flops and 0.085M parameter count. These results indicate that our proposed MKA-Net can address the detection problem of pests with cluster distribution and tiny scales with minimal computation burden.

### 5.5. Exploration of Cut-Up Factors λ

**Figure 15:**
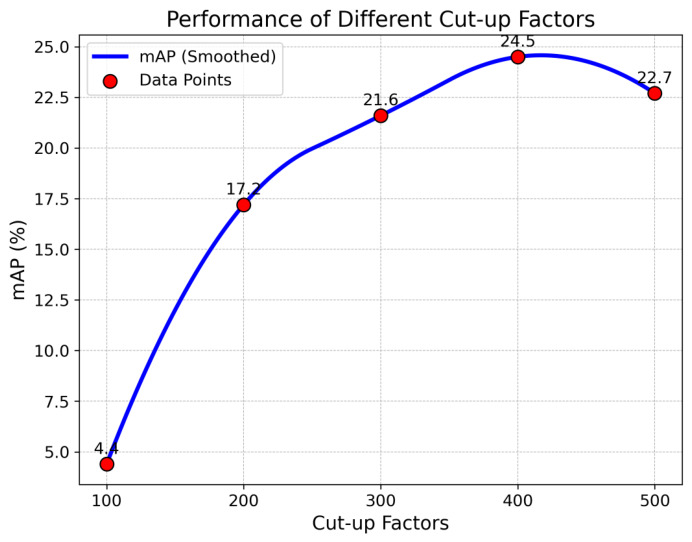
Performance metrics at different cut-up factors.

The proposed cut-up algorithm only has one hyper-parameter, that is, the cut-up factor, which plays an essential role by deciding the split parts of the aggregated image. We have conducted several experiments to obtain the most optimal one. Figure 15 shows the changes in mAP with the cut-up factor, λ. We can observe that the mAP first increases and then drop gradually as λ increases, suggesting that an optimal split exists for pest images. The poor results at small λs is explained by the fact that there are fewer targets in each image, hindering the detection of pests with clustered distribution. A larger λs also degraded the performance of the module. Thus, in this paper, the cut-up factor is set to 400.

### 5.6. Effects of Different Loss Functions

**Figure 16:**
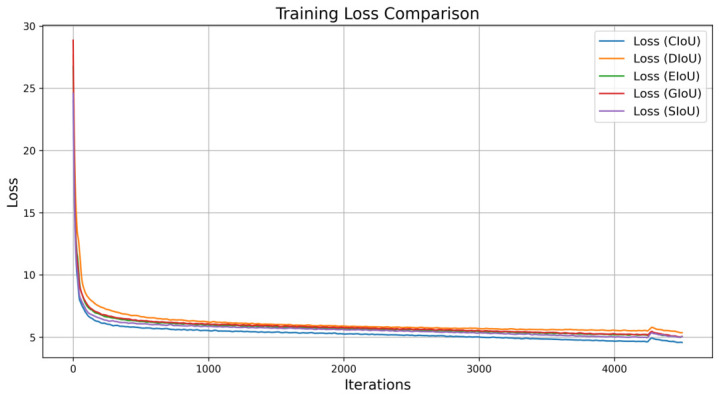
Loss decline plots for different loss functions.

Ultimately, we investigated the impact of different bounding box loss functions on the proposed module. Table 6 indicates that using CIoU as the bounding box loss function significantly enhances accuracy compared to DIoU [33], EIoU [43], GIoU [44], and SIoU [45], with the mAP value reaching a high of 30.8%. Furthermore, we observed that during actual training, CIoU converges more quickly and stably as is shown in Figure 16. Based on this, we have chosen to employ CIoU as the loss function in this paper.

## 6. Conclusions and Future Work

In this paper, we propose an object detection model for densely distributed small-scale pests. Initially, due to the lack of a high-quality dataset for wheat pests, we spent two years collecting and establishing our wheat pest dataset, which more accurately reflects the conditions in agricultural production. However, because of the difficulty in distinguishing pests from complex backgrounds in real-world scenarios, our method proposes the cut-up strategy to separate pest targets from intricate backgrounds. Experiments on multiple classic models show that our cut-up strategy significantly improves pest detection. Furthermore, considering the dense and small-scale nature of pests, we propose the MKA-Net to aggregate, extract, and reconstruct the multi-scale recognizable features of pests. Ultimately, our method achieves an AP50 of 67.1%, outperforming other methods significantly. Nevertheless, the experimental results also reveal that the detection accuracy for severely overlapping wheat pests needs improvement. In the future work, we envision that incorporating entomological priors, like specifically the habits and spatial distribution of pests, could further elevate model performance. By bridging biological knowledge with deep learning architectures, we aim to address complex scenarios more effectively.

## Figures and Tables

**Table 1:** Details of wheat pest dataset.

Pest Species	The Number of Images	The Number of Instances
*Macrosiphum avenae* (F., 1775) (MA)	2668	25,322
*Sipha maydis* Passerini, 1860 (SM)	2600	28,621
*Penthaleus major* (Dugès, 1834) (PM)	284	471
*Rhopalosiphum padi* (Linnaeus, 1758) (RP)	133	369
Total number	5685	54,783

**Table 2:** Number of instances per image at different pixel resolutions.

Number of Instances Per Sheet	100 × 100	200 × 200	300 × 300	400 × 400
0–5	48,678	23,990	16,084	11,984
6–10	2107	2586	2664	2655
11–15	166	522	650	746
16–20	19	107	184	233
>20	3	32	96	133

**Table 3:** Performance of state-of-the-art detection methods on the dense and tiny pest dataset. The best results are in bold.

Model	mAP	mAP50	mAP75	mAP_s_	mAP_m_	mAP_l_	GFlops	Params(M)
Cascade-Rcnn	21.2	56.8	10.2	16.2	25.6	22.2	20.9	69.161
Dynamic-Rcnn	19.4	51.2	9.4	14.1	23.0	20.8	18.2	41.753
Cascade-RPN	21.8	59.1	10.3	18.5	25.3	25.1	16.8	41.952
RT-DETR	23.8	60.9	12.3	16.8	28.5	35.2	32.0	20
FCOS	15.3	40.6	6.6	11.1	18.8	18.2	17.1	32.12
TOOD	21.8	56.6	11.1	16.3	25.7	34.6	17.2	32.025
YOLOX	23.5	59.2	11.7	16.0	26.8	31.0	13.3	8.939
YOLOV5	21.1	54.2	10.5	15.0	24.5	33.3	7.9	7.03
YOLOV6	22.7	58.6	11.4	14.9	26.5	38.7	21.9	17.189
YOLOV7	18.2	50.9	7.5	11.9	21.7	20.3	**6.6**	6.023
YOLOV8	24.8	62.0	13.1	17.3	28.9	32.9	14.3	11.137
YOLOV10	18.0	47.0	8.4	11.5	21.3	21.5	8.2	**2.696**
YOLOV26	23.7	57.8	20.5	10.2	26.4	36.8	20.5	9.466
Proposed method	**30.8**	**67.1**	**22.0**	**20.8**	**37.4**	**39.8**	13.553	9.024

**Table 4:** Ablation studies of using cut-up strategy. The best results are in bold.

Model	mAP	mAP50	mAP75	mAP_s_	mAP_m_	mAP_l_
Cascade-Rcnn	21.2	56.8	10.2	16.2	25.6	22.2
Cascade-Rcnn with cut up	25.7	57.7	17.3	29.5	28.6	20.3
Dynamic-Rcnn	19.4	51.2	9.4	14.1	23.0	20.8
Dynamic-Rcnn with cut up	23.7	55.7	12.8	**26.5**	26.7	16.4
Cascade-RPN	21.8	59.1	10.3	18.5	25.3	25.1
Cascade-RPN with cut up	26.0	57.6	18.8	18.5	29.3	23.1
FCOS	15.3	40.6	6.6	11.1	18.8	18.2
FCOS with cut up	23.5	58.9	11.9	20.8	26.3	20.6
TOOD	21.8	56.6	11.1	16.3	25.7	34.6
TOOD with cut up	26.2	58.4	18.2	18.2	29.2	22.4
YOLOV5	21.1	54.2	10.5	15.0	24.5	33.3
YOLOV5 with cut up	21.9	50.8	15.7	20.4	25.3	1.8
YOLOV6	22.7	58.6	11.4	14.9	26.5	**38.7**
YOLOV6 with cut up	28.7	61.4	22.2	24.5	32.2	24.7
YOLOV7	18.2	50.9	7.5	11.9	21.7	20.3
YOLOV7 with cut up	20.7	49.6	14.2	20.2	22.5	0.9
YOLOV8	24.8	62.0	13.1	17.3	28.9	32.9
YOLOV8 with cut up	29.4	61.5	**23.3**	23.3	32.0	28.9
YOLOV10	18.0	47.0	8.4	11.5	21.3	21.5
YOLOV10 with cut up	25.5	54.5	22.3	18.9	31.3	19.7
YOLOX	23.5	59.2	11.7	16.0	26.8	31.0
YOLOX with cut up	**30.2**	**67.7**	19.6	23.6	**34.9**	31.1

**Table 5:** Performance of proposed module by using cut-up strategy and MKA-Net.

Model	mAP	mAP50	mAP75	mAP_s_	mAP_m_	mAP_l_	GFlops	Params(M)
YOLOX	23.5	59.2	11.7	16.0	26.8	31.0	13.322	8.939
YOLOX with cut up	30.2	67.7	19.6	23.6	34.9	31.1	13.322	8.939
YOLOX with cut up and MKA—Net	30.8	67.1	22.0	20.8	37.4	39.8	13.553	9.024

**Table 6:** Ablation studies of loss functions.

Loss Function	mAP	mAP50
DIoU	29.5	67.2
EIoU	28.5	66.8
GIoU	29.9	68.4
SIoU	29.6	67.2
CIoU	30.8	67.1

## Data Availability

Due to the confidentiality agreement, this dataset cannot be fully disclosed temporarily. However, if there is a research need, the researchers can contact the author via email to obtain it.
